# Oxygen Deprivation Modulates EGFR and PD-L1 in Squamous Cell Carcinomas of the Head and Neck

**DOI:** 10.3389/fonc.2021.623964

**Published:** 2021-02-26

**Authors:** Sebastian Zahnreich, Senayit Gebrekidan, Gabriele Multhoff, Peter Vaupel, Heinz Schmidberger, Arnulf Mayer

**Affiliations:** ^1^ Department of Radiation Oncology and Radiation Therapy, University Medical Center Mainz, Mainz, Germany; ^2^ Radiation Immuno-Oncology Project Group, Central Institute for Translational Cancer Research, Klinikum rechts der Isar, Technische Universität München, Munich, Germany; ^3^ Department of Radiation Oncology, Klinikum rechts der Isar, Technische Universität München, Munich, Germany

**Keywords:** head and neck cancer, epidermal growth factor receptor, Ras-MAPK pathway, PI3K/AKT, PD-L1, hypoxia, apoptosis, autophagy

## Abstract

Abundance and signaling of the epidermal growth factor receptor (EGFR) and programmed cell death protein ligand 1 (PD-L1) in head and neck squamous cell carcinoma (HNSCC) are not only genetically determined but are also subject to the traits of the tumor microenvironment, which has hitherto not been clarified completely. We investigated the impact of hypoxia on the EGFR system and on PD-L1 in six HPV negative HNSCC cell lines *in vitro* and in FaDu xenografts *in vivo*. Protein levels of EGFR, AKT, pAKT, ERK1/2, pERK1/2, CA IX, cleaved PARP (apoptosis), LC3B (autophagy), and PD-L1 were quantified by western blot after oxygen deprivation or CoCl_2_, staurosporine, and erlotinib treatment. In FaDu xenograft tumors the expression of EGFR, CA IX andCD34 staining were analyzed. Reduced oxygen supply strongly downregulated EGFR protein levels and signaling in FaDu cells *in vitro* and *in vivo*, and a transient downregulation of EGFR signaling was found in three other HNSCC cell lines. PD-L1 was affected by oxygen deprivation in only one HNSCC cell line showing increased protein amounts. The results of this study indicate a significant impact of the traits of the tumor microenvironment on crucial molecular targets of cancer therapies with high clinical relevance for therapy resistance and response in HNSCC.

## Introduction

Head and neck squamous cell carcinomas (HNSCCs) of the oral cavity, oropharynx, hypopharynx, or larynx are the sixth most common cancers worldwide accounting for more than 550,000 new cases and 380,000 deaths per year with a predicted increase (1). Most HNSCCs are diagnosed in locally or regionally advanced stages and are treated by multimodal therapies including radical surgery, adjuvant radiotherapy (RT)/platinum-based chemoradiotherapy (CRT), or primary CRT as the standard of care (2). The vast majority of advanced HNSCCs are characterized by resistance to conventional CRT, loco-regional recurrence, and distant metastases (R/M setting) associated with poor patient outcomes and 5-year overall survival rates of about 60% (3). The most prominent oncogenic driver in HNSCC is the epidermal growth factor receptor (EGFR), a transmembrane receptor tyrosine kinase that is expressed or over-expressed in more than 90% of all HNSCCs (4). EGFR mediates tumorigenicity and resistance to CRT by fostering DNA repair, anti-apoptotic signaling, cell growth, and proliferation *via* activation of the Ras/Raf–mitogen-activated protein kinase (Ras-MAPK) pathway, the phosphatidylinositol-4,5-bisphosphate 3-kinase (PI3K)/AKT pathway, and the Janus kinases/signal transducers and activators of transcription (JAK/STAT) pathway (5). High tumor EGFR expression in HNSCC patients correlates with elevated local recurrence and inferior disease-free survival (4). Promising therapeutic strategies have been developed to target EGFR in HNSCC by monoclonal antibodies or small-molecule tyrosine kinase inhibitors (6) that showed limited clinical efficacy due to very heterogeneous and low response rates or the development of drug resistance (7). Currently, clinical trials investigate the therapeutic benefit of immune checkpoint inhibitors, such as antibodies targeting programmed cell death protein 1 (PD-1) and its ligand PD-L1 in the primary and R/M setting for HNSCC (8). HNSCCs are generally considered as an immunologically inert malignancy and more than 60% of HNSCCs overexpress PD-L1 (9). Immune checkpoint inhibitor therapies showed durable improvements in outcomes of HNSCC patients, but as for EGFR-targeted therapies, response rates are very low and tumor cells frequently acquire immunosuppressive resistance to the cytotoxic activity of immune effectors (10–13). Beyond an inhibition of the EGFR-signaling cascade, anti-EGFR IgG1-antibodies induce antibody-dependent, cell-mediated cytotoxicity (ADCC) in HNSCC and, therefore, act as an anti-cancer immunotherapeutic agent itself (14). This offers great potential for a combination of anti-EGFR IgG1-antibodies with immune checkpoint inhibitors in a multimodal setting to amplify the anti-cancer immune response, and to increase response rates and the duration of the response (14, 15).

The EGFR downstream signaling cascades themselves have been shown to regulate PD-L1 expression in different tumor entities (16–19) including HPV negative HNSCC tumor specimens that rely on a tumor-intrinsic, EGFR-directed PD-L1 expression for immune evasion (20). Thus, downregulation of the EGFR-axis by targeted therapies or *via* traits of the tumor microenvironment can prevent PD-L1 upregulation in tumor cells and enhance their immunogenicity. The latter is of high relevance since the molecular phenotype of neoplastic cell populations in HNSCCs is not merely a function of their genetic constitution. It is also determined by the tumor microenvironment or by oncologic therapies. This fact is of central importance for all forms of targeted and immune checkpoint inhibitor therapies since the target structures of these approaches can be induced, degraded, or modified, particularly in the setting of combined modality treatments. E.g., several experimental and clinical studies have shown that the expression of immunosuppressive PD-L1 can be induced by CRT or by hypoxia-induced and inflammatory factors in the tumor microenvironment - as a negative feedback mechanism - to prevent excessive antitumoral inflammatory responses (10, 21–23). In previous work, we have demonstrated that the expression of EGFR is downregulated in diffusion-limited, hypoxic areas of the tumor microenvironment of HNSCCs (24). Tumor hypoxia itself is an adverse prognostic factor for patient outcomes since it promotes tumor progression and resistance to CRT and immune checkpoint inhibitors (11, 25). The additional impact of the hypoxic tumor microenvironment on the expression of molecular targets of oncologic therapies, e.g., the EGFR and its downstream signaling cascades or PD-L1 illustrates the urgent need to investigate such complex interactions to improve the therapeutic response in HNSCC. Concerning the central role of EGFR and PD-L1 in the pathogenesis and therapy of HNSCC, hypoxia-mediated modulation of receptor tyrosine kinase signaling or the immunologic status might represent another paradigm for therapy resistance mediated by tumor microenvironmental traits.

## Materials and Methods

### Cell Culture

Experiments were performed using the following HPV negative HNSCC cell lines: FaDu (kindly provided by Professor Jürgen Brieger, Department of Otolaryngology, Head and Neck Surgery, University Medical Center Mainz, Mainz, Germany), SCC-25 (ATCC, Manassas, USA), SCC-263 [kindly provided by Professor Johan Nuyts, Faculty of Medicine at KULeuven, Leuven, Belgium, originating from (26)], Cal-33, SCC-131 and SCC-9 (kindly provided by Professor Kirsten Lauber and Dr. Michael Orth, Department of Radiation Oncology, University Hospital, Ludwig-Maximilians-Universität München, Munich, Germany). FaDu, Cal-33, and SCC-131 cells were cultured in DMEM low glucose (Merck, Darmstadt, Germany) containing 1% non-essential amino acids (NEA, Merck, Darmstadt, Germany), 15% fetal bovine serum (FBS, Merck, Darmstadt, Germany) and 1% penicillin/streptomycin (P/S, Merck, Darmstadt, Germany). SCC-25 cells were cultured in a 1:1 mixture of DMEM low glucose and Ham’s F-12 medium (Merck, Darmstadt, Germany) containing 1% NEA, 10% FBS, and 1% P/S. SCC-263 cells were cultured in DMEM high glucose (Life Technologies, Carlsbad, USA) containing 1% NEA, 10% FBS, and 1% P/S. SCC-9 cells were cultured in a 1:1 mixture of DMEM low glucose and Ham’s F-12 medium containing 15mM HEPES (Merck, Darmstadt, Germany), 0.5mM sodium pyruvate (Merck, Darmstadt, Germany), 10μM hydrocortisone (Merck, Darmstadt, Germany), 10% FBS and 1% P/S. Cells were maintained in a humidified incubator at 5% CO_2_ and 37°C. Detailed information on HNSCC cell lines is provided in **Supplementary Table S1**.

### Treatment of Cells

For all treatments, cells were seeded and maintained in a humidified incubator at 5% CO_2_ and 37°C for 24 h before the experimental conditions were applied. For mild hypoxia (1% O_2_) cells were transferred to a low-oxygen incubator prepared according to the protocol of Wright and Shay (27). In brief, cell culture dishes were placed in gas-tight containers that were flushed with a well-defined gas mixture consisting of 1% O_2_, 5% CO_2,_ and 94% N_2_ and were placed in an incubator at 5% CO_2_ and 37°C for 48 h. Anoxia (<0.01% O_2_) was induced by using the Anaerocult^®^ A-System (Merck, Darmstadt, Germany) in gas-tight containers for 24 and 48 h. Anoxic conditions were monitored through the Anaerotest^®^-System (Merck, Darmstadt, Germany). Normoxic HNSCC cells were cultured under atmospheric oxygen (21% O_2_) at 5% CO_2_ and 37°C. Treatments with CoCl_2_ (Merck, Darmstadt, Germany) at 150 µM for 48 h, staurosporine (Merck, Darmstadt, Germany) at 1 µM for 2, 4, and 6 h and erlotinib (Cell Signaling Technology, Danvers, USA) at 1 µM for 72 h was conducted at atmospheric oxygen at 5% CO_2_ and 37°C.

### Proliferation and Cell Cycle Analysis

43,000 cells/cm^2^ were seeded and treatments were performed as described above. After treatment, cells were detached by trypsin, and cell numbers were assessed using a MoxiZ cell counter (GeminiBio, Sacramento, USA). Population doublings (PDs) were calculated as described previously (28) out of the number of seeded (N_0_) and harvested (N) cells according to the formula: $\text{PD}=\frac{\text{ln}(N/N_{0})}{ln_{2}}$. For subsequent cell cycle analysis, cells were fixed in 70% Ethanol at -20°C, treated with RNaseA (Merck, Darmstadt, Germany), and stained with HOECHST33258 (Merck, Darmstadt, Germany). At least 10,000 cells per sample were analyzed in a BD FACSCanto II flow cytometer (BD Biosciences, Billerica, USA). Quantification of the fractions of cells in G0/G1, S, and G2/M was performed using the Flowing Software (flowingsoftware.btk.fi). At least three independent experiments with biological triplicates were performed.

### Western Blot

27,000 cells/cm^2^ were seeded and treatments were performed as described above. After treatment, cells were washed with phosphate-buffered saline, scratched from the cell culture dish, lysed in radioimmunoprecipitation assay buffer (Merck, Darmstadt, Germany), and supplemented with a protease inhibitor cocktail (Merck, Darmstadt, Germany). 20µg of denatured proteins were separated by 4–15% sodium dodecyl sulfate (SDS)-polyacrylamide gel electrophoresis and blotted onto a polyvinylidene difluoride membrane (Millipore, Burlington, USA). Membranes were incubated with the following primary antibodies: EGFR, AKT(pan), phospho-AKT(S473), phospho-p44/42 MAPK (Erk1/2, T202/Y204), p44/42 MAPK (Erk1/2), LC3B, PD-L1 (all obtained from Cell Signaling Technology, Danvers, USA), CA IX (Novusbio, Littleton, USA), PARP1 (Abcam, Cambridge, UK), ß-Actin (Thermo Fisher Scientific, Waltham, USA) and horseradish peroxidase (HRP)-conjugated goat anti-rabbit IgG (Amersham™, Little Chalfont, UK). After treatment with the HRP-conjugated antibody, chemiluminescence signals were detected using the Fuji LAS 3000 (Fuji, Minato, Japan), and quantitative measurement of protein expression based on the integrated optical intensity of the chemiluminescence signals was performed using the FIJI software (National Institutes of Health, Berthesda, USA). To enable precise and accurate densitometric quantification of signals from both low and high expression proteins from the same blot and to prevent overloading of the membrane, we have confirmed the linear dynamic range of protein loading for various target proteins in different HNSCC cell lines for our methodology. The data are provided as standard curves of band density *versus* protein load in **Supplementary Figures S1** and **S2**. For multiple subsequent detections, the membranes were stripped using a mild stripping buffer (15g/l Glycin, 1g/L SDS, 10% Tween 20, pH 2.2) and reprobed. Obtained values are shown as relative protein amounts after normalization to the loading control ß-Actin and untreated, normoxic cells. At least three independent experiments were performed.

### Apoptosis (AnnexinV-FITC/PI Staining)

For the assessment of apoptosis, different cell densities were chosen to obtain monolayers at 50% or 100% confluency and to compensate for the growth-limiting effect of oxygen deprivation. Cells were seeded at densities of 35,000 or 140,000/cm^2^ for cultivation under normoxic or hypoxic conditions or at densities of 70,000 or 280,000/cm^2^ for cultivation under anoxia. After the treatments that were performed as described above the cells were harvested by trypsinization and the fraction of apoptotic cells was assessed using an AnnexinV-FITC/PI detection kit (Abcam, Cambridge, UK) according to the manufacturer’s instruction. At least 10,000 cells per sample were measured in a BD FACSCanto II flow cytometer (BD Biosciences, Billerica, USA). The data were analyzed using the Flowing Software (flowingsoftware.btk.fi). At least three independent experiments with biological triplicates were performed.

### Immunohistochemistry and Analysis

Histological sections of FaDu xenograft tumors (n = 5) were kindly provided by Professor Gabriele Multhoff (Radiation Immuno-Oncology Group, Central Institute for Translational Cancer Research, Klinikum rechts der Isar, Technical University Munich, Munich, Germany). Immunofluorescence staining for the antigens CA IX, EGFR and CD34 was carried out according to a procedure established in our laboratory, as published previously (24). In brief, after cutting 4-µm-thick sections with high precision microtomes, specimens were incubated at 60°C for 1 h and deparaffinized in a descending alcohol series. Pretreatment IHC was carried out using antigen-demasking buffers specific for the chosen antigen in each staining round (details are given in **Supplementary Table S2**). Antibody incubation took place either overnight at 4°C or for 1 h at 30°C, depending on the requirements for the specific antigen (**Supplementary Table S2**). Primary antibody detection was carried out using appropriate secondary antibodies bound to an HRP–conjugated polymer (VECTOR ImmPRESS, Vectorlabs, Burlingame, USA). Fluorescence tagging was finally carried out using a palette of fluorescyl-tyramide reagents (**Supplementary Table S2**). Multiplexing of up to 3 antigens (in two series of stains) was achieved by quenching the peroxidase activity of the previous step using additional rounds of heat pretreatment. Buffers appropriate for the antigen, which was going to be investigated in the subsequent round of staining, were used in a modified version of the original protocol published by Toth and Mezey (29). Additionally, all specimens were counterstained with DAPI at a concentration of 2.5µg/mL for 5min (Thermo Fisher Scientific, Waltham, USA). After rinsing with phosphate-buffered saline, samples were covered with a coverslip using a fluorescence mounting medium (Agilent Technologies, Santa Clara, USA). Single‐cell‐based analyses were carried out using the DAPI channel for the segmentation of cell nuclei in the open-source whole slide image analysis software QuPath0.2.0 (https://qupath.github.io/ (30)). Segmentation was followed by the subclassification of cells in tumor cells, stromal cells, and cells located in overt necrotic areas using QuPath’s machine learning features and user-defined examples. Classified single cell-measurements were then exported to a file, and imported in R (31). Plots shown in **Figure 4** were generated using the package “ggplot2”, and linear correlation coefficients were calculated using the base R correlation function.

### Statistical Analysis

Data handling, plotting, and statistics were done using SigmaPlot Version 14 (Systat Software, San Jose, USA) unless stated otherwise. All quantitative data are presented as mean values and standard deviations. For comparison of the means of two or more groups, the Student’s t-test or the one-way analysis of variance (ANOVA) was used, respectively. All levels of significance were set at *p < 0.05*.

## Results

### Proliferation Activity and Cell Cycle Distribution of HNSCC Cell Lines

**Figure 1:**
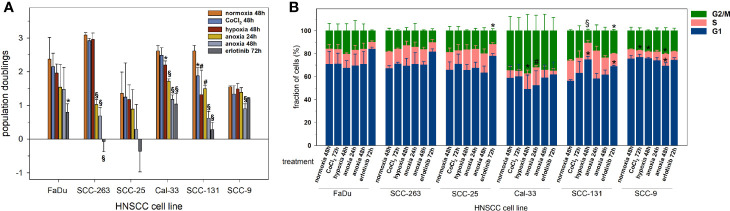
Proliferation activity of six HNSCC cell lines according to **(A)** the accumulation of population doublings, and **(B)** the cell cycle distribution upon normoxia (21% O_2_, 48 h), CoCl_2_ (150 µM, 48 h), mild hypoxia (1% O_2_, 48 h), anoxia (<0.01% O_2_, 24 and 48 h) and erlotinib treatment (1 µM, 72 h). Negative population doublings correspond to cell loss, i.e. after the experiment fewer cells were counted than were plated. Data are shown as mean values and standard deviations of at least 3 independent experiments performed with biological triplicates. Statistics were performed by one way ANOVA, *p < 0.05, ^#^p < 0.01, ^§^p < 0.001.

To evaluate the cytostatic effects of the various treatments applied in this experimental study on exponentially growing HNSCC cell lines, the population doublings were determined up to 72 h after treatment and are shown in **Figure 1A**. CoCl_2_ and mild hypoxia had only mild impacts on the proliferation activity of HNSCC cells with significant decrements in Cal-33 und SCC-131 cells. Anoxia and erlotinib had strong growth-inhibitory effects in all HNSCC cell lines except for SCC-9 cells which showed resistance and normal proliferation activity for all treatments except for prolonged anoxia. The overall cell cycle distribution was comparable between all HNSCC cell lines (**Figure 1B**). Only Cal-33 cells showed an inherent, about twice as high proportion of cells in G2/M compared to all other HNSCC lines. All treatment conditions had only moderate and no systematic effects on the cell cycle distribution in HNSCC cells.

### Viability of HNSCC Cell Lines After Oxygen Deprivation

**Figure 2:**
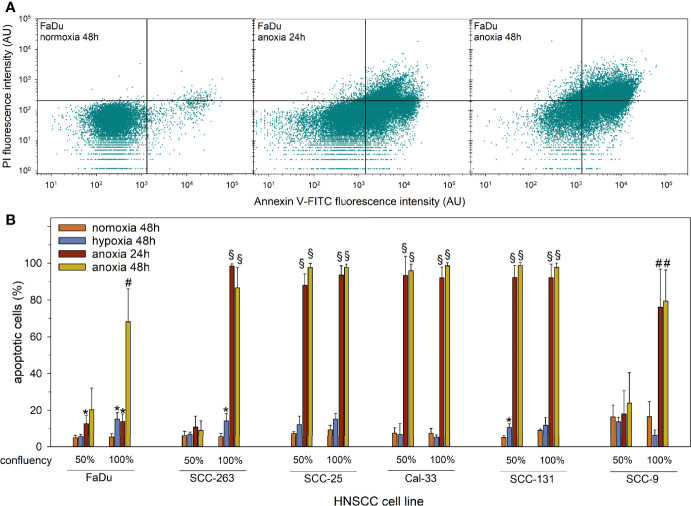
Apoptosis in subconfluent or confluent HNSCC cells upon normoxia (21% O_2_, 48 h), mild hypoxia (1% O_2_, 48 h), or anoxia (<0.01% O_2_, 24 and 48 h) assessed by AnnexinV-FITC/PI staining and flow cytometry. **(A)** Representative dot plots of the measurement of AnnexinV-FITC and PI fluorescence intensities in FaDu cells treated with anoxia at full confluency. Vital cells are shown in the lower left quadrant (AnnexinV-FITC- and PI-), early apoptotic cells (AnnexinV-FITC+ and PI-) are shown in the lower right quadrant, and late apoptotic cells (AnnexinV-FITC+ and PI+) are shown in the upper right quadrant. **(B)** The fractions of apoptotic (total AnnexinV-FITC+) HNSCC cells are shown as mean values and standard deviations of at least three independent experiments performed with biological triplicates. Statistical comparison to normoxic cells was performed by students’ t-test, *p < 0.05, ^#^p < 0.01, ^§^p < 0.001.

To investigate the impact of oxygen deprivation on the viability of HNSCC cells we measured the induction of apoptosis in subconfluent and confluent HNSCC cell lines after 48 h of mild hypoxia or after 24 and 48 h of anoxia. Representative raw data of flow cytometry measurements are shown in **Figure 2A**.

In accordance with a low impact on proliferation activity, treatment with mild hypoxia for 48 h caused only a slight increase in apoptotic cell counts in confluent FaDu and SCC-263 cells, and subconfluent SCC-131 cells. After treatment with anoxia, we observed very variable apoptotic responses depending on cell density and duration of treatment between the six HNSCC cell lines (**Figure 2B**). SCC-263, SCC-25, and Cal-33 cells showed strong and comparable induction of apoptosis in more than 80% of cells for all treatment conditions. For FaDu, SCC-263, and SCC-9 cells the apoptotic response was dependent on cell density and the duration of treatment. In a subconfluent state during anoxia, these cells displayed no or only a mild increase in the fraction of apoptotic cells whereas a steep increment was observed for confluent SCC-263 and SCC-9 cells regardless of the duration of treatment. Even at high cell densities, FaDu cells proved to be largely resistant to anoxia-mediated cell death up to 24 h of treatment. Together, these data show resistance of all investigated HNSCC cell lines to mild oxygen deprivation but very strong variations in their sensitivity to severe anoxia. For the subsequent experiments to study the EGFR system and PD-L1, low cell densities were chosen to prevent the induction of apoptosis under anoxic conditions.

### Oxygen Deprivation Modulates EGFR and PD-L1 in HNSCC

**Figure 3:**
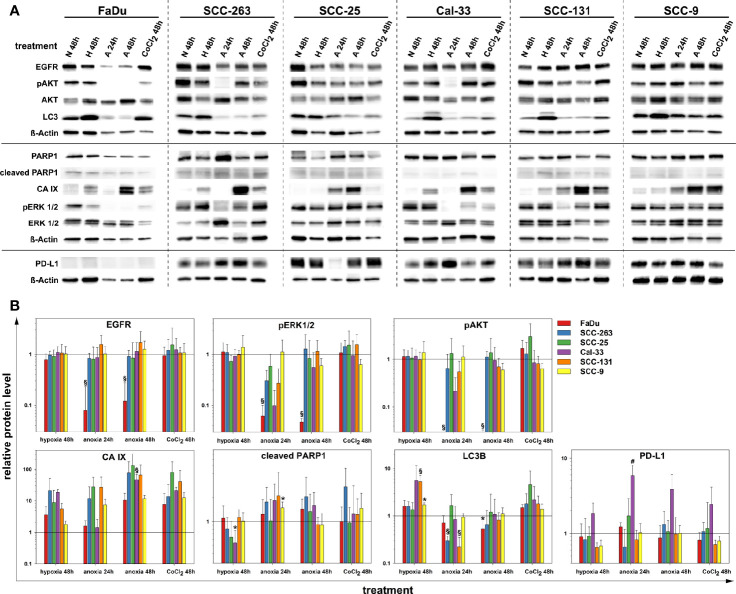
Protein quantification of EGFR, pAKT (S473), AKT (pan), LC3B, PARP1, CA IX, pERK 1/2 (T202/Y204), ERK 1/2, PD-L1 and ß-Actin upon normoxia (N, 21% O_2_, 48 h), mild hypoxia (H, 1% O_2_, 48 h), anoxia (**A**, <0.01% O_2_, 24 and 48 h) and CoCl_2_ (150 µM, 48 h) in six HNSCC cell lines. Results are shown as **(A)** representative western blots, and **(B)** as relative protein levels after normalization to the level of ß-Actin related to normoxic cells. Relative protein levels are provided as mean values and standard deviations of at least three independent experiments performed with one biological sample. Statistics were performed by one way ANOVA, *p < 0.05, ^#^p < 0.01, ^§^p < 0.001.

**Figure 4:**
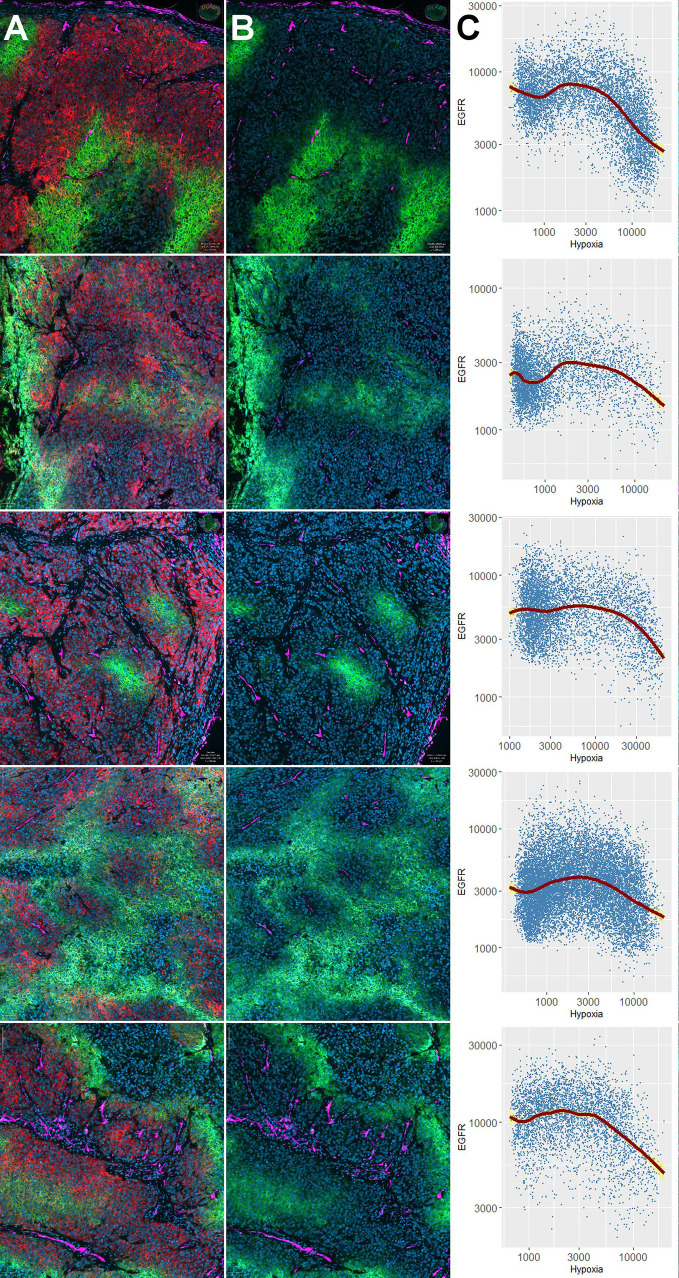
Downregulation of EGFR in hypoxic tissue areas *in vivo*. Distribution of EGFR and carbonic anhydrase (CA) IX in five different FaDu xenograft tumors. Results of individual tumors 1–5 are shown in different rows of the Figure. Panel **(A)** shows 4-plex staining for DAPI (blue, cell nuclei), EGFR (red), CA IX (green), and CD34 (magenta). Panel **(B)** shows the same three markers as in **(A)**, except that the red channel (EGFR) was switched off to illustrate that EGFR-positive areas are negative for CA IX. Note that the hypoxia marker CA IX is partially expressed directly adjacent to blood vessels (e.g., non-functional microvessels), and partially in a typical halo-like fashion at a distance from the nearest microvessel (e.g., 4th row). Panels **(C)** show the results of the single cell-based analysis carried out in QuPath in the form of scatterplots with geometric smoothing (red line), as carried out in R using the “ggplot2” package. The yellow areas correspond to the 95% confidence band for the regression curves. Numeric results for all specimens unanimously show downregulation of EGFR (i.e., negative correlations) with increasing expression of CA IX (R values for Pearson’s product-moment correlation ranged between -0.03 and -0.43, p < 2.2 × 10^-16^ for all specimens).

Next, we examined the impact of oxygen deprivation and CoCl_2_ treatment on the protein abundance of EGFR and the activity of its downstream signal cascades as well as the protein level of PD-L1 by western blot. **Figure 3** shows the analysis of EGFR protein level, the activity of the associated Ras-MAPK (ERK1/2), and PI3K/AKT signaling cascade, the expression of carbonic anhydrase (CA) IX as a cellular (surrogate) marker of hypoxia, poly(ADP-ribose)-polymerase 1 (PARP1) cleavage as a marker of apoptosis, microtubule-associated protein 1 light chain 3 beta (LC3B) as a marker of autophagy and the immune effector PD-L1. According to our results on the viability of HNSCC cells after anoxia at different cell densities (**Figure 2B**), we applied low seeding densities to prevent anoxia-mediated cell death as confirmed by an absence of PARP cleavage in all cell lines for all treatment conditions. Significant and about 10-fold downregulation of EGFR protein level and deactivation of the associated signaling cascades was observed in FaDu cells after 24 and 48 h of anoxia. According to the phosphorylation status, the activity of ERK1/2 was also reduced 10-fold and that of AKT was completely abolished by anoxia in FaDu cells. Also, Cal-33, SCC-131, and SCC-263 cells displayed a transient downregulation of the EGFR signaling cascades, primarily of the phosphorylation of ERK1/2 after 24 h of anoxia without any changes in the protein level of EGFR itself (**Figure 3**). This temporal response was often accompanied by a lack of hypoxia-induced CA IX expression and lower protein levels of LC3B. However, recovery of protein levels and reconstitution of PI3K-AKT and Raf-MAPK signaling occurred at 48 h after anoxia. In SCC-25 and SCC-9 cells, the EGFR protein level and the corresponding signaling cascades were not affected by any treatment. Mild hypoxia and CoCl_2_ induced the protein expression of CA IX and a mild increment of the autophagy-marker LC3B in all HNSCC cell lines but had no impact on the protein level of EGFR and its downstream signaling. PD-L1 was detected at the protein level in all HNSCC cells except for FaDu cells. Only Cal-33 cells showed an impact of reduced oxygen supply by increased protein amounts of PD-L1. Consistent with our findings on FaDu cells after limited oxygen supply *in vitro*, the downregulation of EGFR was present in 5 FaDu xenograft tumors investigated by multiplex IHC (**Figure 4**).

### Impact of Apoptosis and Tyrosine Kinase Inhibition on EGFR and PD-L1 in HNSCC

**Figure 5:**
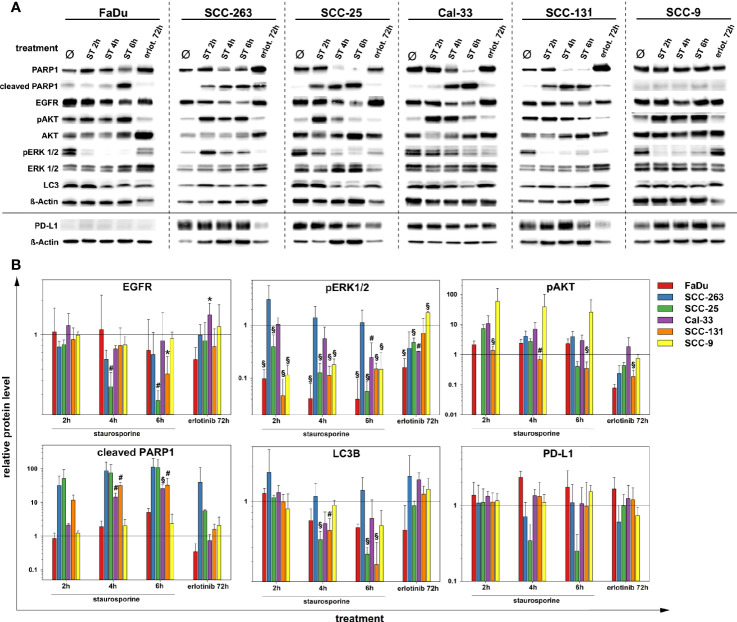
Protein quantification of PARP1, EGFR, pAKT (S473), AKT(pan), pERK 1/2 (T202/Y204), ERK 1/2, LC3B, PD-L1 and ß-Actin after treatment with staurosporine (ST, 1 µM) up to 6 h or erlotinib (1 µM, 72 h) in six HNSCC cell lines. Results are shown as **(A)** representative western blots and **(B)** as relative protein levels after normalization to the level of ß-Actin related to untreated cells. Relative protein levels are provided as mean values and standard deviations of at least 3 independent experiments performed with one biological sample. Statistics were performed by one way ANOVA, *p < 0.05, ^#^p < 0.01, ^§^p < 0.001.

Besides the experimental scenarios of limited oxygen supply, the clinically relevant effects of the global ATP-competitive kinase inhibitor and apoptosis-inductor staurosporine or the tyrosine kinase inhibitor erlotinib on the level of the target proteins and signaling pathways introduced in the previous section were analyzed in HNSCC cells by western blot (**Figure 5**). As confirmed by PARP cleavage, staurosporine readily induced apoptosis in SCC-263, SCC-25, Cal-33 and SCC-131 cells whereas FaDu and SCC-9 cells showed a delayed or no response, respectively. An early reaction to apoptosis-induction in HNSCC cells was the phosphorylation of AKT. This was most pronounced and persistent in FaDu and SCC-9 cells consistent with their highest resistance to apoptosis induced by staurosporine or anoxia (**Figure 2B**). All other cell lines showed a time-dependent decline in the initial AKT phosphorylation. In contrast to the staurosporine-mediated increase in AKT-phosphorylation, a general downregulation of ERK1/2-phosphorylation was found except for SCC-263 cells. Prolonged exposure to staurosporine caused a significant downregulation of the EGFR protein level in SCC-25 and SCC-131 cells. Similarly, the protein abundance of LC3B showed an overall slight reduction after extended staurosporine treatment, again most pronounced in SCC-25 and SCC-131 cells. For PD-L1, decreasing protein amounts were observed during prolonged apoptosis-induction in SCC263 and SCC25 cells, albeit not to a significant extent. The treatment of HNSCC cell lines with erlotinib did not cause gross changes in the protein level of EGFR but affected its downstream signaling cascades. Downregulation of ERK1/2 phosphorylation was observed in FaDu, SCC-25, and Cal-33 cells. The PI3K/AKT signaling pathway was significantly compromised in FaDu, SCC-263, SCC-25, and SCC-131 cells. Only Cal-33 cells showed signs of an apoptotic response after erlotinib treatment as detected by PARP1 cleavage. No significant impact of erlotinib on autophagic activity, measured as LC3B protein levels, or on PD-L1 was found in all HNSCC cell lines.

## Discussion

EGFR has received much attention and is still a pivotal molecular target in HNSCC. However, the modulation of EGFR and the associated signaling pathways by features of the tumor microenvironment, in particular by hypoxia, has not been investigated systematically. This also applies to promising target structures of immuno-oncology such as PD-L1. The paradigm of an alteration of receptor tyrosine kinase signaling or immune effectors by hypoxia is of clinical relevance in the setting of multimodal oncologic strategies, not only in HNSCC (23, 32). Previously, we demonstrated the downregulation of EGFR protein levels in diffusion-limited and mostly CA IX-positive areas of HNSCC tumor specimens (24). The present study was conducted to affirm our hypothesis that tumor hypoxia negatively modulates EGFR protein abundance and activity which might alter the tumor response upon combined modality treatments. Besides, we monitored the protein expression of PD-L1 as an important immune suppressor and mediator of cancer immune escape. Our previous findings were supported by a strong downregulation of EGFR protein level and its signaling cascades under anoxic conditions in FaDu cells *in vitro* and *in vivo*. Moreover, transient anoxia-mediated downregulation of the EGFR-triggered Ras-MAPK- and PI3K/AKT-pathway, albeit with unchanged EGFR protein levels, was confirmed in 3 HNSCC cell lines (Cal-33, SCC-131, and SCC-263) *in vitro*. PD-L1 was affected by limited oxygen availability in just one HNSCC cell line (Cal-33) by increased protein amounts.

Reduced oxygen supply has been shown to have an impact on EGFR and its signal cascades and has been related to insistence on anti-EGFR strategies in various tumor cell lines both grown in culture or as xenograft tumors as well as in human HNSCC tumors including our previous work (24, 33–39). However, the outcomes of these studies are controversial whether hypoxia mediates elevated or diminished protein levels and activity of EGFR. Franovic et al (33). demonstrated that hypoxia is a physiological stimulus that induced EGFR overexpression in a panel of human cancer cell lines *via* activation of the hypoxia-inducible factor 2α (HIF-2α). A HIF-2α-mediated activation of the EGFR signaling pathway including PI3K/AKT by hypoxia was also described by Wang and Schneider (34) in 2 out of 5 EGFR-overexpressing HNSCC cell lines *in vitro* and within hypoxic foci in human HNSCC tumors. In contrast, the comprehensive work of the Nijmegen group demonstrated that EGFR-expression is present mainly in oxygenated areas of HNSCC tumor specimens near blood vessels (35–38). Remarkably, pAKT was detected at longer distances from the vessels in hypoxic and EGFR-negative areas suggesting that the PI3K/AKT-pathway can be activated by hypoxia in an EGFR-independent manner in these tumors. This observation was confirmed by the authors in HNSCC cell lines after short-term hypoxia treatment *in vitro*. However, limited oxygen supply and EGFR-expression were not always mutually exclusive since EGFR was also found in some hypoxic tumor areas. In the present study, an inverse relationship between EGFR and pAKT was detected in apoptotic HNSCC cell lines but has not been confirmed *in vivo*. In a subsequent study, Keulers et al. (39) showed an increased EGFR membrane localization but unchanged expression levels during hypoxia *in vitro* in various cancer cells and HNSCC tumor specimens. The authors identified a role for the hypoxia-inducible GABA_A_ receptor-associated protein like1 protein in the trafficking of EGFR to the plasma membrane, indicating an activation of the EGFR signaling pathway in hypoxic tumor areas. It should be noted that these mostly immuno-histopathological detections primarily considered the expression and localization of the EGFR at the cell membrane whereas the western blot technique applied in the present study also includes *de novo* synthesized or internalized receptors in all cellular compartments. Downregulation of cell surface receptors, including EGFR, occurs as an early and general response after activation through ligand-stimulated endocytosis and subsequent intracellular lysosomal degradation (40), a process that is also dependent on oxygen supply (41). Recently, the paralleling studies of Garvalov et al. (42) and Hentze et al. (43) identified the hypoxia-inducible prolyl hydroxylase domain-3 (PHD3) as a novel regulator of EGFR-signaling and internalization in glioblastoma. Increased PHD3 levels led both to a strong suppression of EGFR-mediated proliferation and a pronounced increase in apoptosis. For EGFR, however, non-degenerative internalization and cytosolic or nuclear accumulation with persistent kinase activity also play a crucial role in treatment resistance and therapeutic success (44).

Degradation of EGFR can also occur through autophagy, a lysosomal pathway and cellular self-degradation process that is activated by cellular stressors such as hypoxia *via* the HIF-1/BCL2/adenovirus E1B 19 kDa protein-interacting protein 3 (BNIP3)–BNIP3L–Beclin1 axis (45) and HIF-1/platelet-derived growth factor receptor signaling (46). In a set of human tumor cell lines and corresponding xenograft tumors, Liu et al. (47) observed a general hypoxia-mediated upregulation of EGFR protein levels at low cell densities whereas amounts of EGFR protein and the activity of the Ras-MAPK- and PI3K/AKT-axis were downregulated in a subset of these cell lines at confluency due to elevated autophagy. In the present study, a hypoxic environment induced autophagy in subconfluent HNSCC cells to variable degrees but was not associated with a change in EGFR protein levels. Also, a decrease in the amounts of EGFR protein under anoxic conditions did not correlate with the level of autophagy in the respective cell line (FaDu). Rather, anoxia was mostly associated with a decrease in autophagic activity. Since oxygen-deprivation has cytostatic effects and can initiate cell death (47), we also examined cellular survival under anoxic conditions in a cell density-dependent manner. The susceptibility to anoxia-mediated apoptosis varied between cell lines and was most pronounced in confluent monolayers. In general, at modest cell densities, all treatments caused mild to moderate cytostatic effects that attenuated proliferation with no major impact on cell cycle distribution or signs of excessive cell death. EGFR plays a crucial role in cell viability by activating the anti-apoptotic PI3K/Akt/mammalian target of rapamycin (mTOR) pathway (48). Intriguingly, FaDu cells showed the most significant downregulation of EGFR and the related signaling pathways under oxygen deprivation but were most resistant to anoxia-mediated apoptosis. Staurosporine, a well-known inducer of apoptosis, had variable, but in general mild effects on the EGFR protein level and triggered the cytoprotective PI3K/AKT-pathway. Conversely, the mitogenic Ras-MAPK pathway was downregulated in five out of six cell lines during the apoptotic response. Treatment with the tyrosine kinase inhibitor erlotinib showed EGFR-dependent proliferation for all cell lines except for SCC-9. Erlotinib compromised the EGFR signaling cascade and attenuated cell proliferation but had no impact on the EGFR level itself, the induction of apoptosis or autophagy in contrast to previous reports by others (49, 50).

Recently, primary resistance to PD-1/PD-L1-targeted immune checkpoint immunotherapy that is observed in more than 80% of HNSCC patients in the R/M setting (12, 13) has been related to intratumoral hypoxia as shown by an inverse spatial distribution of hypoxia- and immune-response markers in HNSCC specimen (10). Tumor hypoxia is a well-known immunosuppressor that compromises T cell and natural killer cell functions, dampens the production of cytokines, impairs cellular metabolism, and upregulation of coinhibitory receptors enabling tumors to evade the host immune response (11). E.g., within such hypoxic ‘immune deserts’, immune escape of cancers can be fostered by HIF-1α-triggered PD-L1 expression (10, 51). However, in our panel of six HNSCC cell lines, upregulation of PD-L1 protein level during oxygen deprivation was found in just one of these cell lines, not indicating a general hypoxia-triggered immune evasion in HNSCC. Although an increment of PD-L1 has already been shown for hypoxic breast and prostate cancer cells *in vitro* (51), the interplay of various factors of the tumor microenvironment or an additional impact of oncologic therapies may be necessary to promote this effect in HNSCC *in vivo* (23). A direct link between the EGFR signaling cascade and PD-L1 expression in HNSCC has been revealed by Concha-Benavente et al. (20). These authors found, that wild-type, overexpressed EGFR significantly correlated with JAK/STAT pathway activation and the induction of PD-L1 expression in a large cohort of HNSCC specimens. Inhibition of the JAK/STAT signaling pathway prevented PD-L1 upregulation in HNSCC cells and enhanced their immunogenicity. Several studies on EGFR-dependent expression of PD-L1 extended the spectrum beyond the JAK/STAT signaling pathway in a variety of human tumor entities. Parsa et al (16). found that loss of the phosphatase and tensin homolog (PTEN) and consequential activation of the PI3K/AKT pathway increased the expression of PD-L1 in glioma. Recently, their finding has been expanded with a role for the EGFR-Ras-MAPK signaling pathway by upregulation of COP9 signalosome complex subunit six (CSN6) and subsequent stabilization of PD-L1 in gliomas (52). In non-small-cell lung carcinoma, EGFR-activating mutations were significantly associated with increased PD-L1 expression that could be downregulated by treatment with erlotinib (17, 18). This was not the case for wild-type EGFR, suggesting that PD-L1 expression is increased by EGFR signaling conferred by activating EGFR mutations (17) and the promotion of immune escape in lung cancer *via* increased PD-L1 expression through resistance to EGFR-directed small tyrosine kinase inhibitors (53). In hepatocellular carcinoma cells, a Ras-MAPK-dependent expression of PD-L1 has also been prevented by the use of small-molecule inhibitors against EGFR and ERK 1 and 2 (19). However, in HPV negative HNSCC cell lines with presumably wild-type EGFR, we could not show any effect of erlotinib treatment on the protein level of PD-L1. Considering these scenarios, a microenvironment-mediated downregulation of EGFR and the associated signaling cascades should lead to a downregulation of PD-L1 increasing tumor immunity and the impact of immuno-oncological therapies. In contrast to a PD-L1 enhancing effect of EGFR-activity, Hu et al. (54) postulated an inverse correlation between AKT or pAKT and PD-L1 expression in lung adenocarcinomas in line with a poor response of patients with EGFR mutations to PD-1/PD-L1 inhibitors. Thus, future studies using primary tumor specimens are highly warranted to unravel the complex interplay of hypoxia, cell-intrinsic EGFR-signaling, and PD-L1 expression to optimize multimodal antineoplastic strategies.

## Conclusion

We demonstrate an impact of oxygen deprivation, a crucial trait of the tumor microenvironment, on a receptor kinase system exemplarily using EGFR and the associated PI3K/Akt and Ras-MAPK signaling cascades as well as on the immune effector PD-L1 in HNSCC. Our findings are of high clinical relevance, not only for anti-EGFR- and anti-PD-L1-directed cancer therapies but also for a broad spectrum of molecular target structures of multimodal oncologic strategies. Such complex interactions between different traits of the tumor microenvironment (e.g., hypoxia, glucose deprivation, and acidosis) and molecular targets of anti-cancer therapies warrant more in-depth explorations to ultimately increase therapeutic efficacy in the treatment of HNSCC and other tumor entities.

## Data Availability Statement

The raw data supporting the conclusions of this article will be made available by the authors, without undue reservation.

## Ethics Statement

The animal study was reviewed and approved by District Government of Upper Bavaria, Maximilianstr. 39., 80538 Munich, Germany.

## Author Contributions

Conception and design: AM, SZ, and HS. Acquisition of data: SZ, SG, and AM. Analysis and interpretation of data: SZ, SG, and AM. Initial draft of the manuscript: SZ. Writing, review, and/or revision of the manuscript: SZ, AM, GM, PV, SG, and HS. All authors contributed to the article and approved the submitted version.

## Funding 

This study was conducted with intramural funding of the University Medical Centre Mainz, Germany.

## Conflict of Interest

The authors declare that the research was conducted in the absence of any commercial or financial relationships that could be construed as a potential conflict of interest.

## Supplementary Material

Supplementary Table 1 Characteristics of HNSCC cell lines.

Supplementary Table 2 Antibodies (AB), immunohistochemical techniques, and resulting staining patterns.

Supplementary Figure 1 Densitometric quantification of serial dilutions from 2.5, 5, 10, 15, 20, 25, and 30 µg of total protein lysate per lane from **(A)** SCC-9, **(B)** FaDu and **(C)** SCC-131 cells. The following proteins were detected by western blot: EGFR, PARP1, phospho-p44/42 MAPK (Erk1/2, T202/Y204), p44/42 MAPK (Erk1/2), phospho-AKT(S473), AKT(pan), LC3B, PD-L1, and ß-Actin. Linear regression models are fitted to the data as shown by straight lines with 95% confidence intervals (CI).

Supplementary Figure 2
**(A)** Correlations of the data on densitometric quantification of target proteins from serial protein dilutions of FaDu, SCC-9, and SCC-131 cells (**Supplementary Figure S1**) and the loading control ß-Actin. Linear regression models are fitted to the data as shown by straight lines. **(B)** Ratio of the intensity of the protein bands of target proteins and ß-Actin. Data are shown as mean and standard deviation from FaDu, SCC-9, and SCC-131 cells. Except for a mild overestimation of the protein levels of PARP1 and EGFR at a low concentration of 5 µg loaded protein, overall very good correlations of target proteins and the loading control ß-actin were obtained.

## References

[1] Marur S Forastiere AA. Head and Neck Squamous Cell Carcinoma: Update on Epidemiology, Diagnosis, and Treatment. Mayo Clin Proc (2016) 91(3):386–96. 10.1016/j.mayocp.2015.12.017 26944243

[2] Colevas AD Yom SS Pfister DG Spencer S Adelstein D Adkins D. NCCN Guidelines Insights: Head and Neck Cancers, Version 1.2018. J Natl Compr Cancer Network: JNCCN (2018) 16(5):479–90. 10.6004/jnccn.2018.0026 29752322

[3] Belcher R Hayes K Fedewa S Chen AY. Current treatment of head and neck squamous cell cancer. J Surg Oncol (2014) 110(5):551–74. 10.1002/jso.23724 25053506

[4] Ang KK Berkey BA Tu X Zhang HZ Katz R Hammond EH. Impact of epidermal growth factor receptor expression on survival and pattern of relapse in patients with advanced head and neck carcinoma. Cancer Res (2002) 62(24):7350–6. 12499279

[5] Oda K Matsuoka Y Funahashi A Kitano H. A comprehensive pathway map of epidermal growth factor receptor signaling. Mol Syst Biol (2005) 1:2005 0010. 10.1038/msb4100014 PMC1681468 16729045

[6] Harari PM Allen GW Bonner JA. Biology of interactions: antiepidermal growth factor receptor agents. J Clin Oncol (2007) 25(26):4057–65. 10.1200/JCO.2007.11.8984 17827454

[7] Tian Y Lin J Tian Y Zhang G Zeng X Zheng R. Efficacy and safety of anti-EGFR agents administered concurrently with standard therapies for patients with head and neck squamous cell carcinoma: a systematic review and meta-analysis of randomized controlled trials. Int J Cancer (2018) 142(11):2198–206. 10.1002/ijc.31157 29143328

[8] von der Grun J Rodel F Brandts C Fokas E Guckenberger M Rodel C. Targeted Therapies and Immune-Checkpoint Inhibition in Head and Neck Squamous Cell Carcinoma: Where Do We Stand Today and Where to Go? Cancers (2019) 11(4):472. 10.3390/cancers11040472 PMC6521064 30987257

[9] Ferris RL. Immunology and Immunotherapy of Head and Neck Cancer. J Clin Oncol (2015) 33(29):3293–304. 10.1200/JCO.2015.61.1509 PMC4586169 26351330

[10] Brooks JM Menezes AN Ibrahim M Archer L Lal N Bagnall CJ. Development and Validation of a Combined Hypoxia and Immune Prognostic Classifier for Head and Neck Cancer. Clin Cancer Res (2019) 25(17):5315–28. 10.1158/1078-0432.CCR-18-3314 31182433

[11] Chouaib S Noman MZ Kosmatopoulos K Curran MA. Hypoxic stress: obstacles and opportunities for innovative immunotherapy of cancer. Oncogene (2017) 36(4):439–45. 10.1038/onc.2016.225 PMC5937267 27345407

[12] Ferris RL Blumenschein G Jr. Fayette J Guigay J Colevas AD Licitra L. Nivolumab for Recurrent Squamous-Cell Carcinoma of the Head and Neck. N Engl J Med (2016) 375(19):1856–67. 10.1056/NEJMoa1602252 PMC5564292 27718784

[13] Mehra R Seiwert TY Gupta S Weiss J Gluck I Eder JP. Efficacy and safety of pembrolizumab in recurrent/metastatic head and neck squamous cell carcinoma: pooled analyses after long-term follow-up in KEYNOTE-012. Br J Cancer (2018) 119(2):153–9. 10.1038/s41416-018-0131-9 PMC6048158 29955135

[14] Trivedi S Srivastava RM Concha-Benavente F Ferrone S Garcia-Bates TM Li J. Anti-EGFR Targeted Monoclonal Antibody Isotype Influences Antitumor Cellular Immunity in Head and Neck Cancer Patients. Clin Cancer Res (2016) 22(21):5229–37. 10.1158/1078-0432.CCR-15-2971 PMC5093040 27217441

[15] Mazorra Z Chao L Lavastida A Sanchez B Ramos M Iznaga N. Nimotuzumab: beyond the EGFR signaling cascade inhibition. Semin Oncol (2018) 45(1-2):18–26. 10.1053/j.seminoncol.2018.04.008 30318080

[16] Parsa AT Waldron JS Panner A Crane CA Parney IF Barry JJ. Loss of tumor suppressor PTEN function increases B7-H1 expression and immunoresistance in glioma. Nat Med (2007) 13(1):84–8. 10.1038/nm1517 17159987

[17] Azuma K Ota K Kawahara A Hattori S Iwama E Harada T. Association of PD-L1 overexpression with activating EGFR mutations in surgically resected nonsmall-cell lung cancer. Ann Oncol: Off J Eur Soc Med Oncol (2014) 25(10):1935–40. 10.1093/annonc/mdu242 25009014

[18] Akbay EA Koyama S Carretero J Altabef A Tchaicha JH Christensen CL. Activation of the PD-1 pathway contributes to immune escape in EGFR-driven lung tumors. Cancer Discov (2013) 3(12):1355–63. 10.1158/2159-8290.CD-13-0310 PMC3864135 24078774

[19] Xing S Chen S Yang X Huang W. Role of MAPK activity in PD-L1 expression in hepatocellular carcinoma cells. J BUON (2020) 25(4):1875–82. 33099927

[20] Concha-Benavente F Srivastava RM Trivedi S Lei Y Chandran U Seethala RR. Identification of the Cell-Intrinsic and -Extrinsic Pathways Downstream of EGFR and IFNγ That Induce PD-L1 Expression in Head and Neck Cancer. Cancer Res (2016) 76(5):1031–43. 10.1158/0008-5472.CAN-15-2001 PMC4775348 26676749

[21] Sato H Jeggo PA Shibata A. Regulation of programmed death-ligand 1 expression in response to DNA damage in cancer cells: Implications for precision medicine. Cancer Sci (2019) 110(11):3415–23. 10.1111/cas.14197 PMC6824998 31513320

[22] Jiang X Wang J Deng X Xiong F Ge J Xiang B. Role of the tumor microenvironment in PD-L1/PD-1-mediated tumor immune escape. Mol Cancer (2019) 18(1):10. 10.1186/s12943-018-0928-4 30646912 PMC6332843

[23] Ock CY Kim S Keam B Kim S Ahn YO Chung EJ. Changes in programmed death-ligand 1 expression during cisplatin treatment in patients with head and neck squamous cell carcinoma. Oncotarget (2017) 8(58):97920–7. 10.18632/oncotarget.18542 PMC5716702 29228662

[24] Mayer A Zahnreich S Brieger J Vaupel P Schmidberger H. Downregulation of EGFR in hypoxic, diffusion-limited areas of squamous cell carcinomas of the head and neck. Br J Cancer (2016) 115(11):1351–8. 10.1038/bjc.2016.336 PMC5129814 27802455

[25] Vaupel P Mayer A. Hypoxia in cancer: significance and impact on clinical outcome. Cancer Metastasis Rev (2007) 26(2):225–39. 10.1007/s10555-007-9055-1 17440684

[26] Begg AC Sprong D Balm A Martin JM. Premature chromosome condensation and cell separation studies in biopsies from head and neck tumors for radiosensitivity prediction. Radiother Oncol (2002) 62(3):335–43. 10.1016/S0167-8140(01)00498-4 12175565

[27] Wright WE Shay JW. Inexpensive low-oxygen incubators. Nat Protoc (2006) 1(4):2088–90. 10.1038/nprot.2006.374 17487199

[28] Zahnreich S Melnikova L Winter M Nasonova E Durante M Ritter S. Radiation-induced premature senescence is associated with specific cytogenetic changes. Mutat Res (2010) 701(1):60–6. 10.1016/j.mrgentox.2010.03.010 20338260

[29] Tóth ZE Mezey E. Simultaneous visualization of multiple antigens with tyramide signal amplification using antibodies from the same species. J Histochem Cytochem (2007) 55(6):545–54. 10.1369/jhc.6A7134.2007 17242468

[30] Bankhead P Loughrey MB Fernández JA Dombrowski Y McArt DG Dunne PD. QuPath: Open source software for digital pathology image analysis. Sci Rep (2017) 7(1):16878. 10.1038/s41598-017-17204-5 29203879 PMC5715110

[31] Team RC. R: A language and environment for statistical computing. Vienna, Austria: R Foundation for Statistical Computing (2019). Available at: https://wwwR-projectorg/.

[32] Hynes NE MacDonald G. ErbB receptors and signaling pathways in cancer. Curr Opin Cell Biol (2009) 21(2):177–84. 10.1016/j.ceb.2008.12.010 19208461

[33] Franovic A Gunaratnam L Smith K Robert I Patten D Lee S. Translational up-regulation of the EGFR by tumor hypoxia provides a nonmutational explanation for its overexpression in human cancer. Proc Natl Acad Sci (2007) 104(32):13092–7. 10.1073/pnas.0702387104 PMC1941796 17670948

[34] Wang X Schneider A. HIF-2alpha-mediated activation of the epidermal growth factor receptor potentiates head and neck cancer cell migration in response to hypoxia. Carcinogenesis (2010) 31(7):1202–10. 10.1093/carcin/bgq078 PMC2893799 20395290

[35] Nijkamp MM Hoogsteen IJ Span PN Takes RP Lok J Rijken PF. Spatial relationship of phosphorylated epidermal growth factor receptor and activated AKT in head and neck squamous cell carcinoma. Radiother Oncol (2011) 101(1):165–70. 10.1016/j.radonc.2011.06.022 21775008

[36] Hoogsteen IJ Marres HA van den Hoogen FJ Rijken PF Lok J Bussink J. Expression of EGFR under tumor hypoxia: identification of a subpopulation of tumor cells responsible for aggressiveness and treatment resistance. Int J Radiat Oncol Biol Phys (2012) 84(3):807–14. 10.1016/j.ijrobp.2012.01.002 22420963

[37] Stegeman H Kaanders JH Wheeler DL van der Kogel AJ Verheijen MM Waaijer SJ. Activation of AKT by hypoxia: a potential target for hypoxic tumors of the head and neck. BMC Cancer (2012) 12:463. 10.1186/1471-2407-12-463 23046567 PMC3517352

[38] Stegeman H Kaanders JH van der Kogel AJ Iida M Wheeler DL Span PN. Predictive value of hypoxia, proliferation and tyrosine kinase receptors for EGFR-inhibition and radiotherapy sensitivity in head and neck cancer models. Radiother Oncol (2013) 106(3):383–9. 10.1016/j.radonc.2013.02.001 PMC3627829 23453541

[39] Keulers TG Schaaf MB Peeters HJ Savelkouls KG Vooijs MA Bussink J. GABARAPL1 is required for increased EGFR membrane expression during hypoxia. Radiother Oncol (2015) 116(3):417–22. 10.1016/j.radonc.2015.06.023 26164772

[40] Lemmon MA Schlessinger J. Cell signaling by receptor tyrosine kinases. Cell (2010) 141(7):1117–34. 10.1016/j.cell.2010.06.011 PMC2914105 20602996

[41] Wang Y Roche O Yan MS Finak G Evans AJ Metcalf JL. Regulation of endocytosis via the oxygen-sensing pathway. Nat Med (2009) 15(3):319–24. 10.1038/nm.1922 19252501

[42] Garvalov BK Foss F Henze AT Bethani I Graf-Hochst S Singh D. PHD3 regulates EGFR internalization and signalling in tumours. Nat Commun (2014) 5:5577. 10.1038/ncomms6577 25420589

[43] Henze AT Garvalov BK Seidel S Cuesta AM Ritter M Filatova A. Loss of PHD3 allows tumours to overcome hypoxic growth inhibition and sustain proliferation through EGFR. Nat Commun (2014) 5:5582. 10.1038/ncomms6582 25420773 PMC4263145

[44] Dittmann K Mayer C Rodemann HP. Nuclear EGFR as novel therapeutic target: insights into nuclear translocation and function. Strahlentherapie und Onkologie (2010) 186(1):1–6. 10.1007/s00066-009-2026-4 20082181

[45] Zhang H Bosch-Marce M Shimoda LA Tan YS Baek JH Wesley JB. Mitochondrial autophagy is an HIF-1-dependent adaptive metabolic response to hypoxia. J Biol Chem (2008) 283(16):10892–903. 10.1074/jbc.M800102200 PMC2447655 18281291

[46] Wilkinson S O’Prey J Fricker M Ryan KM. Hypoxia-selective macroautophagy and cell survival signaled by autocrine PDGFR activity. Genes Dev (2009) 23(11):1283–8. 10.1101/gad.521709 PMC2701580 19487569

[47] Liu B Han D Zhang T Cheng G Yinliang L Wang J. Hypoxia-induced autophagy promotes EGFR loss in specific cell contexts, which leads to cell death and enhanced radiosensitivity. Int J Biochem Cell Biol (2018) 111:12–8. 10.1016/j.biocel.2018.09.013 30278227

[48] Carnero A Blanco-Aparicio C Renner O Link W Leal JF. The PTEN/PI3K/AKT signalling pathway in cancer, therapeutic implications. Curr Cancer Drug Targets (2008) 8(3):187–98. 10.2174/156800908784293659 18473732

[49] Fung C Chen X Grandis JR Duvvuri U. EGFR tyrosine kinase inhibition induces autophagy in cancer cells. Cancer Biol Ther (2012) 13(14):1417–24. 10.4161/cbt.22002 PMC3542232 22954701

[50] Tsien CI Nyati MK Ahsan A Ramanand SG Chepeha DB Worden FP. Effect of erlotinib on epidermal growth factor receptor and downstream signaling in oral cavity squamous cell carcinoma. Head Neck (2013) 35(9):1323–30. 10.1002/hed.23128 PMC4364542 22907806

[51] Barsoum IB Smallwood CA Siemens DR. Graham CH. A mechanism of hypoxia-mediated escape from adaptive immunity in cancer cells. Cancer Res (2014) 74(3):665–74. 10.1158/0008-5472.CAN-13-0992 24336068

[52] Su L Guo W Lou L Nie S Zhang Q Liu Y. EGFR-ERK pathway regulates CSN6 to contribute to PD-L1 expression in glioblastoma. Mol carcinogenesis (2020) 59(5):520–32. 10.1002/mc.23176 32134157

[53] Peng S Wang R Zhang X Ma Y Zhong L Li K. EGFR-TKI resistance promotes immune escape in lung cancer via increased PD-L1 expression. Mol Cancer (2019) 18(1):165. 10.1186/s12943-019-1073-4 31747941 PMC6864970

[54] Hu ZY Huang WY Zhang L Huang B Chen SC Li XL. Expression of AKT and p-AKT protein in lung adenocarcinoma and its correlation with PD-L1 protein and prognosis. Ann Trans Med (2020) 8(18):1172. 10.21037/atm-20-5865 PMC7576079 33241021

