# Supplementary material for: Oxygen Deprivation Modulates EGFR and PD-L1 in Squamous Cell Carcinomas of the Head and Neck
Source: Front Oncol. 2021 Feb 26;11:623964. doi: 10.3389/fonc.2021.623964 (PMC7953989; doi:10.3389/fonc.2021.623964)

Supplementary Table S1

Cell line	Origin		p53 status
**Cal-33**	Squamous Cell Carcinoma (Tongue)	Established from the surgically removed fragment of a tongue lesion from a 69-year-old man with moderately differentiated squamous cell carcinoma of the tongue in 1983 (prior to therapy).<br>DOI: 10.1016/0277-5379(88)90335-5	Mut. (R175H)
**SCC-9**	Squamous Cell Carcinoma (Tongue)	Established from a tongue squamous cell carcinoma of a 25-year-old caucasian man	c.822_853del32
**SCC-131**	Squamous Cell Carcinoma (oral cavity)	Established from an oral squamous cell carcinoma of a 73-year-old caucasian man	Tyr205Ser (c.614A>C)
**FaDu**	Squamous Cell Carcinoma (pharynx)	Established in 1968 from a punch biopsy of an hypopharyngeal tumor removed from a 56 year old caucasian man	c.376-1G>A and c.743G>T
**SCC-263**	Squamous Cell Carcinoma (oral cavity)	obtained from a biopsy from a head and neck tumor<br><br>https://doi.org/10.1016/S0167-8140(01)00498-4	R306X in exon 8 (CGA.TGA) and a 32-bp deletion in exon 7 (del 704–735)
**SCC-25**	Squamous Cell Carcinoma (tongue)	Established from a tumor removed from a 70 year old caucasian man.	c.625_626delAG

Supplementary Table S2

Antigen	Primary AB (Cat.-No., clone, species, dilution, incubation)	Primary AB supplier	Fluorochrome (Cat.-No.)	Staining pattern
**EGFR**	4267, D38B1, rabbit, 1:50, overnight at 4°C	Cell Signaling Technology, Danvers, MA (USA)	Alexa Fluor 647 Tyramide (T20951)	Membranous
**CA IX**	ab108351, EPR4151[2], rabbit, 1 : 200, 1 h at 28 - 30°C	Epitomics, Burlingame, CA (USA)	Alexa Fluor 488 Tyramide (T20948)	Membranous
**CD34**	ab81289, EP373Y, rabbit, 1:150, for 1 h at 24°C	Abcam, Cambridge (UK)	Alexa Fluor 546 Tyramide (T20923)	Membranous

Supplementary Figure S1

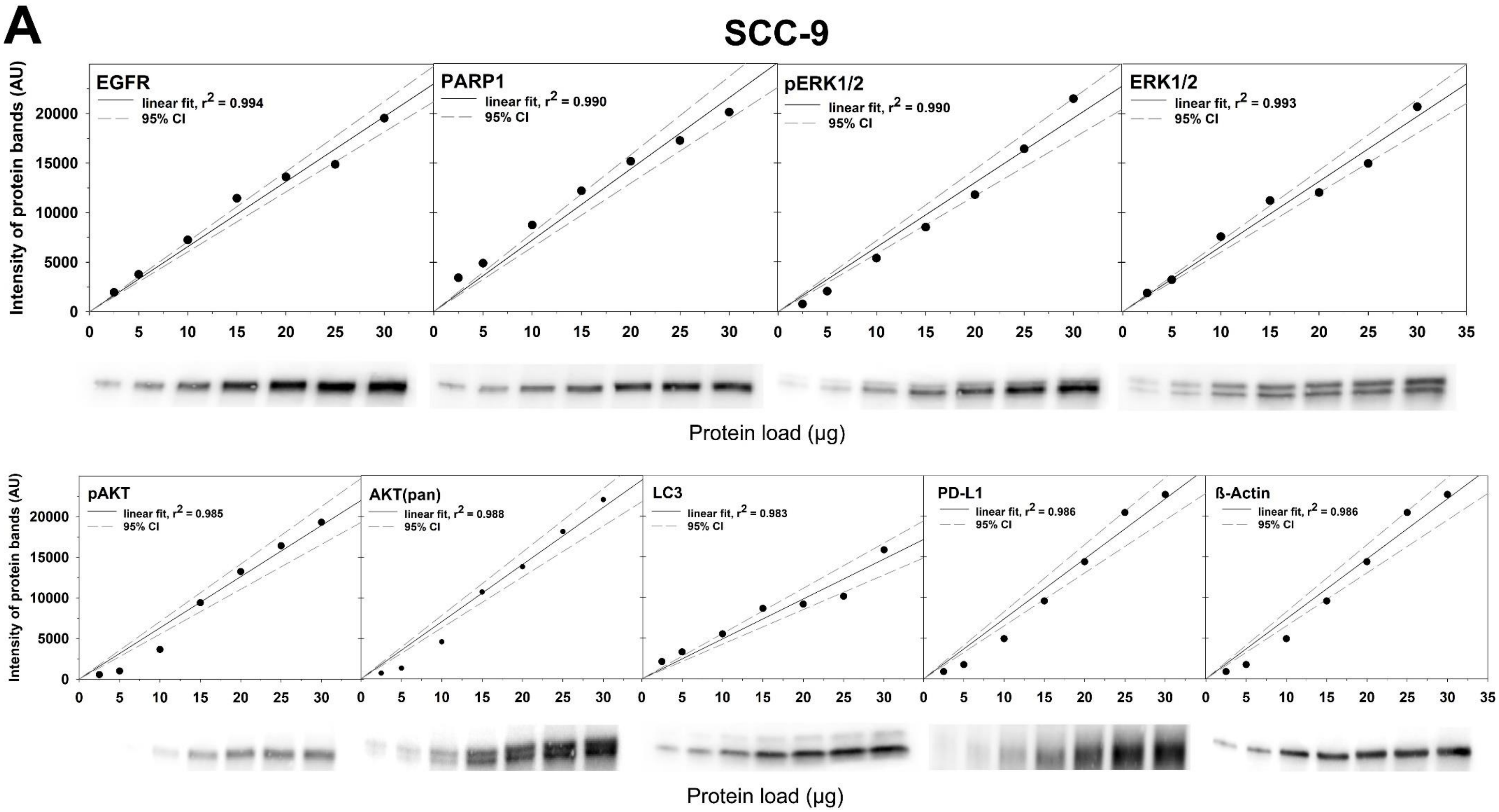

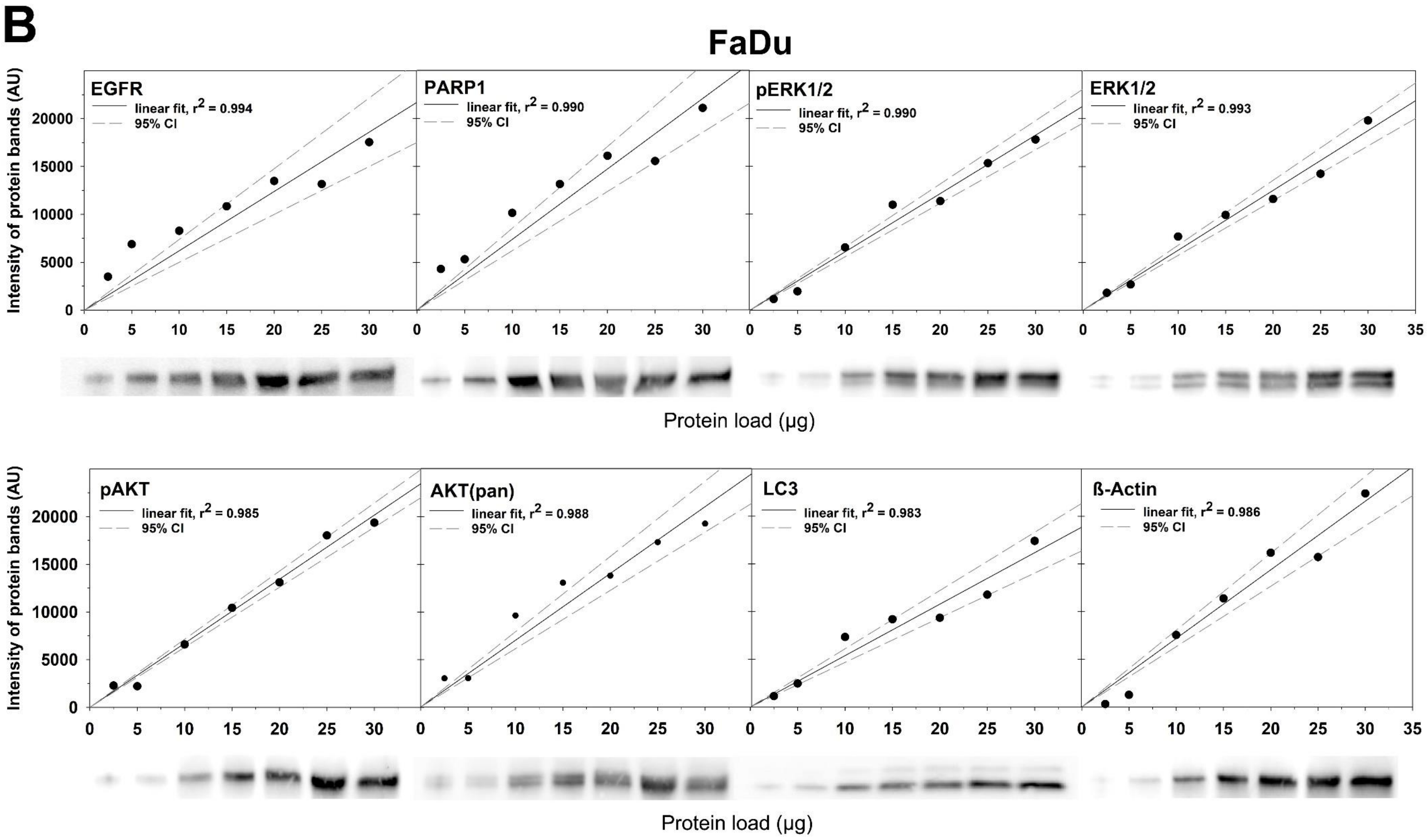
B
FaDu
EGFR
PARP1
pERK1/2
ERK1/2
pAKT
AKT(pan)
LC3
ß-Actin
linear fit, r2 = 0.994
linear fit, r2 = 0.990
linear fit, r2 = 0.990
linear fit, r2 = 0.993
linear fit, r2 = 0.985
linear fit, r2 = 0.988
linear fit, r2 = 0.983
linear fit, r2 = 0.986
95% CI
Intensity of protein bands (AU)
Protein load (µg)

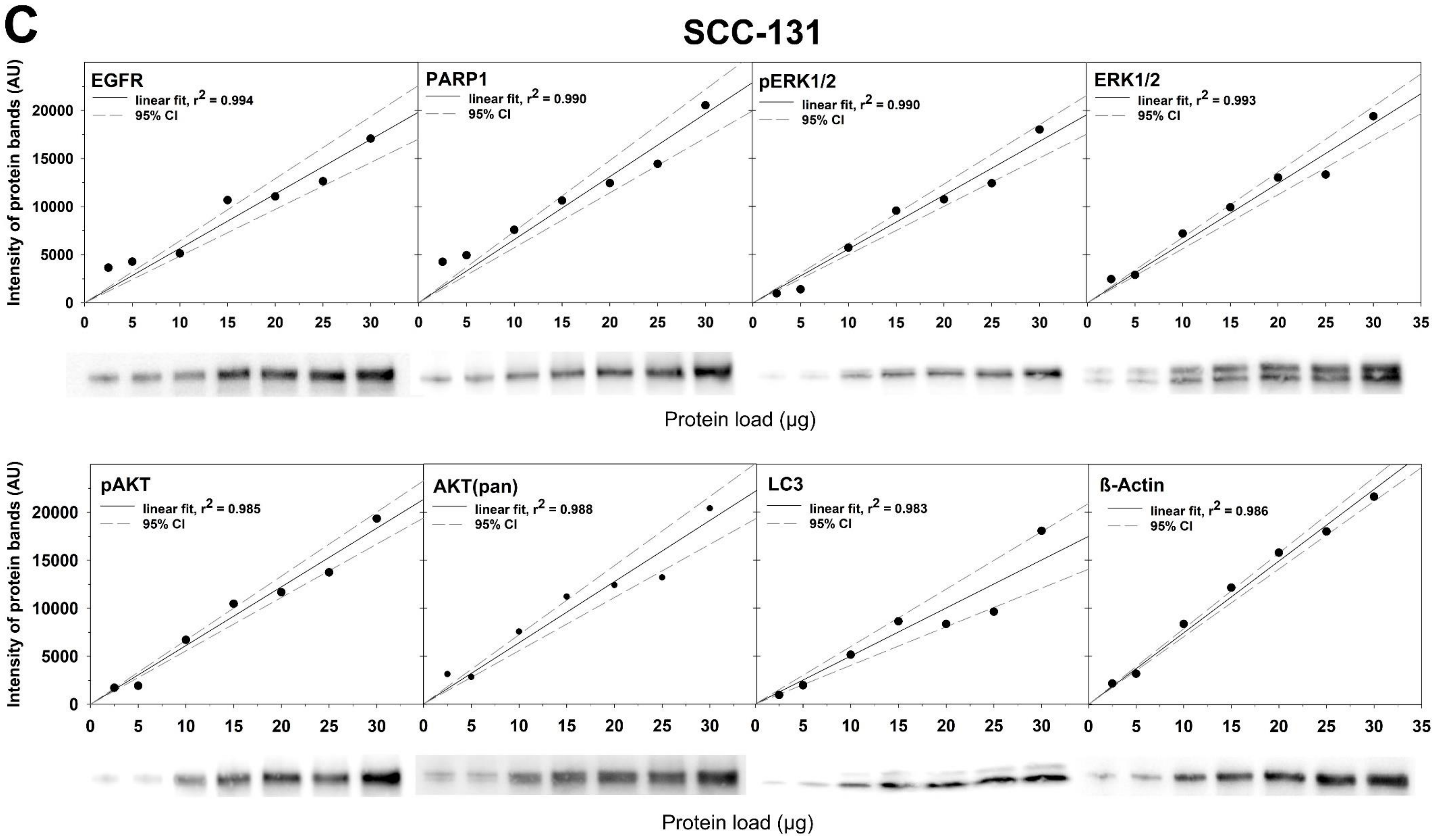

C
SCC-131
Intensity of protein bands (AU)
EGFR
PARP1
pERK1/2
ERK1/2
pAKT
AKT(pan)
LC3
ß-Actin
linear fit, r^2 = 0.994
linear fit, r^2 = 0.990
linear fit, r^2 = 0.990
linear fit, r^2 = 0.993
linear fit, r^2 = 0.985
linear fit, r^2 = 0.988
linear fit, r^2 = 0.983
linear fit, r^2 = 0.986
95% CI
Protein load (µg)

Supplementary Figure S2

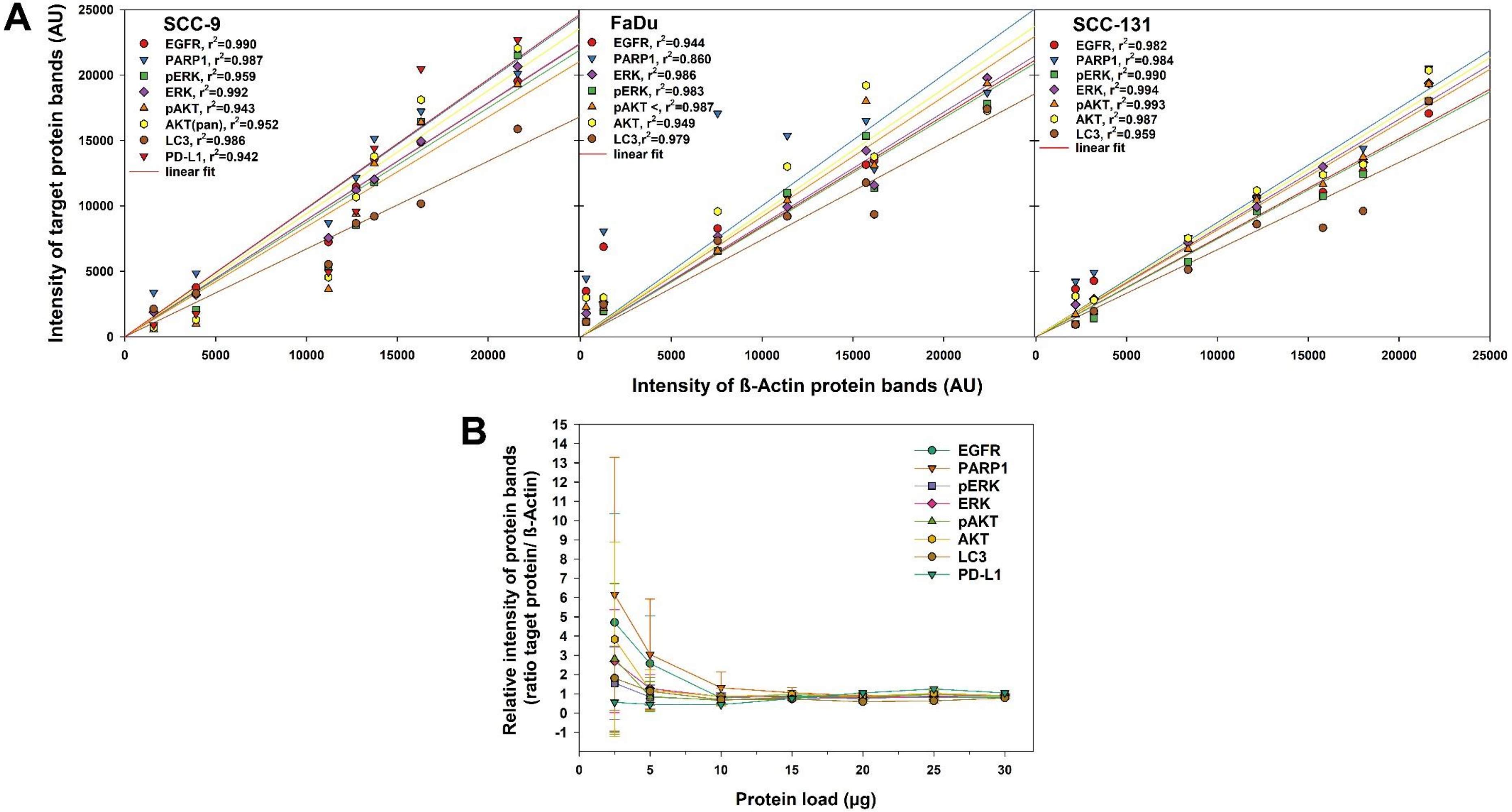

Supplement: Supplementary Table 1 — Characteristics of HNSCC cell lines. [file DataSheet_1.pdf]
